# A Study of Incivility in the Iranian Nursing Training System Based on Educators and Students’ Experiences: A Quantitative Content Analysis

**DOI:** 10.5539/gjhs.v7n2p203

**Published:** 2014-10-28

**Authors:** Mostafa Rad, Es-hagh Ildarabadi, Fatemeh Moharreri, Hossein Karimi Moonaghi

**Affiliations:** 1 Nursing and Midwifery School, Mashhad University of Medical Sciences, Mashhad, Iran; 2 Department of Nursing, Esfarayen Faculty of Medical Sciences, Esfarayen, Iran; 3 Child andAdolescent Psychiatry, Psychiatry and Behavioral Sciences Research Center, Mashhad University of Medical Sciences, Mashhad, Iran; 4 Medical-Surgical Department, Faculty of Nursing and Midwifery, Mashhad, Iran; 5 Medical Education Department, Mashhad University of Medical Sciences, Mashhad, Iran

**Keywords:** incivility, education, nursing, quantitative content Analysis, disrespectful behavior, Iran

## Abstract

**Background:**

It is absolutely essential to know the negative impacts incivility in students and educators may have on the creation of a suitable teaching-learning environment. Better education of to-be nurses would improve their service to patients and society in the future. There has been no research in Iran so far on this particular case. This study examines the experiences of uncivil or disrespectful behavior from the standpoint of educators and students.

**Methodology & Methods:**

A quantitative content analysis was carried out to study manuscripts presented in the form of open questionnaires. To this end, data produced from detailed answers from 640 students and educators were inputted into the computer and line-by-line and sentence-by-sentence coding was done. After that, implied codes were added, the categories were revealed, and finally counting frequency of code in categories was carried out.

**Results:**

The most important categories that students considered uncivil behavior were waste of class time, distraction, incompetence in managing the class, discrimination, bad assessment, insult and threat on behalf of the educators. In contrast to their view, what the educators thought of as disrespectful included class disorder, humiliation of other students, irregular attendance of classes, bad sitting postures, non-observance of Islamic standards, and coming unprepared to the class by students.

**Conclusion:**

From the viewpoint of students and educators, incivility is present towards one another in the academic environment. This study determines the most important forms of the same from their stand point. Since disrespectful and threatening behavior has a significant impact on learning environment, we highly recommend a thorough examination to be carried out in future studies on the origin and the managing strategies of such behaviors.

## 1. Introduction

Teaching is beyond mere transfer of information or skill development. In fact, it is an evolutionary process for both trainer and trainee. Throughout the process, sometimes some problems called disrespectful behavior or incivilities arise that disrupt the interaction between student and educator. In such cases, the educator should be familiar with the problem and is responsible for solving it individually or cooperatively. Having fulfilled this, he/she can enjoy a pleasant experience in the class along with the students. Here some points are worthy of mention (Hubbell & Hubbell, 2010), incivility in nursing training refers to the indiscriminate destructive behavior which brings about physical or mental distress for those involved in the teaching process. If no action is taken to overcome this problem, it may turn out to be a threatening situation (Feldmann, 2001a; Clark, & Carnosso, 2008). One of the consequences of incivility in the class is that bad behavior interferes with a normal learning environment, especially in group learning. Nowadays this kind of behavior is considered to be a serious problem throughout the world (Feldmann, 2001b). It is today’s student who is going to be tomorrow’s colleague; so, not determining and managing their disrespectful behavior will change them into uncivil personnel in the long run (Luparell, 2011). They would grow into people with vulnerable personalities which may do irreversible damage in the future, including problems in interaction with colleagues, patients, and the organization (Karimi Moonaghi, 2014). Disrespectful behavior may lead to significant secondary problems too. These problems relate to patient security, absence from work, service abandonment, workplace movement, organizational commitment, and personnel’s physical and mental health (Lim & Lee, 2011; Felblinger, 2008). More than fifty percent of nurses had experienced disrespectful behavior, and over ninety percent of them had witnessed behavioral abuse in their workplace (Clark et al., 2011). In fact, students’ disrespectful behavior is a growing problem in the academic nursing society (Robertson, 2012; Karimi Moonaghi et al., 2009). This kind of behavior is seen both in students as well as the educators, yet their viewpoints in this regard are different (Feldmann, 2001b; Luparell, 2008). Many researches have been carried out on incivility in other countries, some of which are mentioned here. In Gallo’s view, disrespectful behavior is a serious problem in nursing training. This behavior includes many unpleasant behaviors like class disruption, students’ indifference, disrespect for instructor and/or other classmates, delay in attending the class and leaving early, dishonesty, and bullying. This disrespectful behavior weakens the learning environment and leads to violence in the workplace (Gallo, 2012).

Disrespectful behavior towards students is also deeply offensive so much so that students suffering from the same are mentally and physically affected and see themselves as weak people against problems. Such behavior not only has profound impacts on their self-confidence and independence but also on their bodies (Clark, 2008c), that too when countries are facing shortages of nurses and disorder in health care (Luparell, 2004). Given this fact, determining and reviewing methods effective in checking disrespectful behavior is necessary (Swinney, 2010). Also, research on such behavior in nursing colleges is essential. Students of Medical Sciences universities should be both skillful as well as have a high standard of ethics (Swick, 2000). Professionalism is an inseparable aspect of medical practice and dishonest employees and cheats can endanger society (Hafeez et al., 2013). Unacceptable, unprofessional behavior is seen taking place in both students and educators. Although there have been reviews on incivility in some health-care vocations like nursing, dentistry and pharmacology, but no research on this subject has been undergone in Iran and so to say on Eastern culture, yet (Luparell, 2011, Rowland & Srisukho, 2009; Clark, 2008b). Therefore, students and educators’ experiences in this regard were studied. Since the concept of incivility among students and educators of Iranian society had not been scrutinized, the researchers carried out the study using an open questionnaire with the following questions: 1) as an educator, what is your opinion about incivility of students? 2) As a student, what is your opinion about incivility of educators?

## 2. Methodology & Methods

### 2.1 Design

A non-empirical quantitative content analysis approach was used to analyze open questionnaires that distributed among nursing students and educators in the province of Khorasan Razavi in Iran. Quantitative content analysis was chosen to schematically and objectively describe, classify and count the numerous responses of the students and educators about incivility (Burla et al., 2008).

### 2.2 Participants

The research was done in 2014 and a total of 100 educators and 540 students participated in this study as census and filled out the questionnaires (Table 1).

**Table 1:** Participants’ characteristics

Characteristics		Participants
Educational level and scientific rank(N)*	BSc. Student(500) MSc Student (40)	Assistant Professor(4) Educator(96)
Age(years)	19-35	32-55
Gender(N)	Male(216)	Male(30)
	Female(324)	Female(70)
Experience(years)	-	2-30

* Number.

### 2.3 Data Gathering and Analysis

The researchers carried out the study using an open questionnaire with the following questions: 1) as an educator, what is your opinion about incivility of students? 2) As a student, what is your opinion about incivility of educators? Apart from this goal of the research, another one hypothesized was to find out if there is disrespectful behavior in this society, and if so, to what extent? This could be achieved from the number of similar replies on that subject. The questionnaires were handed to the students and educators. Then, at an appointed time, the filled in questionnaires were taken back. All the educators and students’ responses to the open questions were typed word by word and inputted into the computer. Next, the content was repeatedly readout by researcher and the main themes were extracted based on the question. In this study, all the students were in Bachelor’s or Master’s levels in nursing and their educators had Master’s or Ph.D. degrees.

The responses to open questions were content analyzed with the assistance MAXQDA software program. MAXQDA is a software program designed for coding and interpreting textual data. At first researchers defined the unit of analysis and categories. The unit of analysis in this study was each words, phrases or themes numbered as incivility in responses to open questions. The responses were initially coded at the sentence level with each code assigned in distinct category with similarities in meaning. Primary coding was done by one researcher and was audited by a second researcher. The analysis continued with definition the categories, denotation of rules, examining the reliability of each category and the reviewing of the categories where necessary. In final stage frequency of codes in each category was counted in order to assess the extent of incivility in students and educators.

### 2.4 Trustworthiness

The researchers asked an expert (external auditor) to code each text simultaneously, and what was agreed upon by the two was considered the measure. In other words, apart from the researcher’s comprehension of the data, inter-coder reliability was also enhanced (Cavanagh, 1997).

The reliability and disagreement between inter-coders was assessed by the Cohen’s kappa coefficient (Burla et al., 2008). The Kappa value of 0.41–0.60 was considered as average agreement, more than 0.60 was considered as satisfactory or solid agreement and more than 0.8 meant complete agreement (Everitt, 1996).

The extent of agreement between the two code-givers was more than 80% in all the categories, some of which are mentioned here. The Kappa values for categories of Waste Class Time and Distraction, Incompetence in Managing the Class, Insult and Threat, and Discrimination were 0.97, 0.83, 0.8 and 90%, respectively; these values were considered as complete agreement.

### 2.5 Ethical Considerations

The ethical consideration was approved by ethical committee of the Mashhad University of Medical Sciences. The oral consent was taken from the participants before given the questionnaire. They were assured that their statements would remain confidential.

## 3. Results

The most repeated codes from the students’ point of view settled in 11 category were as follows: 294 cases for the waste of class time and distraction (54%), 288 cases for incompetence in class management (53%), 258 cases for insult and threat (47.8%), 165 cases for discrimination (30.5%), 135 cases for bad assessment (25%), 135 cases for inappropriate communication with students and personnel (25%), 66 cases for dishonesty and lack of commitment (12%), 102 cases for ignoring students’ opinions (19%), 75 cases for mistrust of students (13.8%), 69 cases for non-observance of Islamic standards (12.7%), and 105 cases for high expectation from the students to cater to patients, do their assignments and non-strictness (19.4%).

Also, the most repeated codes from the educators’ point of view settled in 16 category were as follows: 98 cases for disrespect towards educators (98%), 95 cases for class order (95%), 87 cases for humiliating fellow classmates (87%), 70 cases for irregular attendance in class (70%), 63 cases for sitting postures and observance of Islamic standards (63%), 60 cases for their being unprepared for the class (60%), 39 cases for playing with cell phones or sending SMS (39%), 39 cases for blind modeling from others (39%), 30 cases for cheating on homework or in exams (30%), 21 cases for insisting on ending the class before the appointed time and wanting breaks for no reason (21%), 18 cases for preferring extra-curricular activities to the normal formal course (18%), 18 cases for non-observance of ethics towards colleagues or even college peers (18%), 15 cases for non-observance of patients’ rights (15%), 15 cases for wasting public funds and for lack of commitment (15%), 12 cases for bringing unrelated affairs into college (12%), 9 cases for students’ disrespectful behavior towards university staff (9%), 9 cases for non-observance of hospital or clinical regulations (9%), and 3 cases for not caring for patients at the appropriate time (3%).

## 4. Discussion

According to the students’ experiences, wastage of class time and distraction, incompetence in class management, insult, humiliation, disrespect and threat, discrimination, bad assessment and inappropriate communication with students and department staff were among the most important themes. The results of this research bore similarities as well as differences compared to other studies. For example, Feldman classified disrespectful behavior in class environment into 4 categories including irritation (going to class late, leaving early and answering cell phones), classroom terrorism, intimidation and violence (Feldmann, 2001b). As can be seen, despite the fact that irritation is common in both researches, it has not been mentioned as much by the students in assessing educator incivility. Given the other categories of Feldman, it can be seen that these categories are among the most common mentioned behaviors in our research, too.

In Clark’s phenomenological research, *humiliation of students*, *intimidation by educator, and high expectations from students* have been described as incivility. In that study, many students mentioned the misuse of power as the main culprit; so, more focus on this category to find effective solutions will pave the way for a better relationship between educator and student, and lead to a good teaching environment (Clark, 2008a). In the present research, *power abuse* manifested itself as pride in the educators. This differed with other works on the subject done abroad and it could be due to cultural differences in educators studied. In another study by Clark, cancelation of class without informing students, being ill-prepared for the class, restricting discussions, belittling students’ efforts and reproaching them, perfunctory teaching, and unavailability outside the classroom have been reported as incivility on behalf of educators (Clark & Springer, 2007). It seems that there are some commonalities between the current study and Clark’s. In other words, despite cultural differences, there seems to be similarities in the expectations of students from their educators throughout the world (Joybari, 2010a) However, discrimination and bad assessment by the educators have been two themes in the current study that are not found in Clark’s.

On the other hand, from the Iranian educators’ point of view, the most repeated behavior codes were disrespect towards the educator, class disorder, humiliation of other students, students’ irregular attendance, improper sitting postures, non-observance of Islamic standards, and students’ lack of preparedness for class. In a study by Luparell, educators considered 18 behaviors as disruptive; from among these, 100% of the educators looked at latency, absence and distraction as incivility of students. More than half of the participants were dissatisfied with the classroom noise and hubbub as well as with the clinical environment. Also, 24.8 percent of them complained of fighting. Twenty-one percent believed that the amount of incivility has increased compared to 5 years ago (Luparell, 2004). As can be observed, student disorder, which includes latency, absence and distraction, is considered disrespectful act towards the educator and is a common theme in both studies. One difference in our Iranian study and Luparell’s is that physical struggle reported in the latter is not seen in the former, and this can be considered a sign of good behavior in Iran. In a study by Kalantari et al, Medical Science students stated “humiliation of students” as the most significant theme. Other behaviors mentioned by the students were mistrust, lack of praise after achievement, cold behavior, use of inappropriate words, having a bad opinion of students, disrespect towards the field, and being unpunctual, all of which, is to a great extent in line with the present study (Kalantari et al., 2012).

Students and educators had considerably dissimilar opinions about incivility. According to the educators, disrespectful behavior was mostly related to disrespect towards educator, class order, humiliation of other students, irregular attendance at class, sitting position and observance of Islamic standards in class, students’ unpreparedness for class, playing with cellphones or sending SMS, blindly copying others or the environment, and making fun of classmates or mocking the educator while he is answering questions. On the other hand, students had a more comprehensive view of educators’ behaviors; they mentioned bad assessment, incompetence in class management, insult, humiliation, discrimination, waste of class time and distraction, bad rapport with students and employees, non-observance of Islamic standards, high expectation of students in doing homework and patients’ tasks, indifference, ignoring students’ opinions, not being open to criticism, making fun of students, dishonesty, and a lack of commitment. It seems that the educators had a milder criticism of students than vice versa. Most seemingly ignored many of the students’ disrespectful behaviors as they considered it usual for that age, and so did not see these behaviors as a serious obstacle for teaching. In contrast to their views, 51.3 percent of the students considered incivility in the teaching environment as a disruptive factor (Joybari, 2010b). Joybari’s study reveals that only 4 percent of educators mentioned “making fun of educators” as incivility. While 68 percent of the students stated that they had seen such behavior from their educators in the past year. These findings reveal the generation and cultural gap between students and educators. Students are not mature enough and as they are sensitive; they may consider many of their educators’ behaviors as insulting. However, educators do not have such a pessimistic standpoint.

Since educators are role models for their students, it is important to know the expectations students may have of them (Karimi Moonaghi et al., 2009; Gallo, 2012). Thus, by knowing students’ expectations, educators can do their best to change those negative attributes. As for their acceptable behaviors, they should convince students that such behavior is necessary for an enhanced teaching-learning environment. Thus, educators too, improve their behavior to be good models for their students. Also, disrespectful behavior on the part of students mentioned by educators should be considered and thought over in order to find appropriate solutions for the same. The least advantage of these findings is that both students and educators can predict incivility and avoid getting shocked and confused in such situations by adopting preventive actions.

Moreover, some common viewpoints heard from both sides, such as delay, non-observance of Islamic standards and disrespect, reveal that a portion of student incivility is a mere reflection of that of the educators and staff.

To know more about incivility and its mental and social effects, further comprehensive research is needed, and to decrease such behavior in the academic environment, cultural awareness is necessary; otherwise, it can negatively impact the teaching environment (Olender-Russo, 2009). Since the onset of this behavior can be rooted in the teaching environments, the adoption of preventive approaches seems vital (Clark, 2009). The sampling was limited to just one province of Iran (Khorasan Razavi); however, this limitation is considerably removed because the people who teach or study at the colleges of this province come from all parts of Iran. Other limitations include the fact that as the items were written down, each participant had commented on incivility in his/her own way and may have forgotten some other behaviors due to a shortage of time. On the other hand, one advantage of this study was the anonymity of the participants, which enabled them to answer the question with an ease of mind and out of sight of their seniors and researchers.

## 5. Conclusion

Generally, the findings revealed that incivility exists among Iranian students and educators, the most important of which were mentioned by the students and educators. With regards to the same, the necessity of getting more information on such behavior because of its disruptive effects on the learning environment encouraged the researcher to carry out the study. Yet, it is recommended that in future studies, the roots of such behaviors and the solutions be scrutinized via quantitative and qualitative methods so that students and educators can continue their teaching-learning process peacefully. Also, it is recommended that managers hold seminars for educators to examine different aspects of this problem. Obviously, experienced educators can transfer their experience to novices, although this does not make them exempted from getting more knowledge in the field. Finding the roots of such behaviors and management strategies to overcome the problems using a special model is necessary. Using complementary studies, educators can attain a greater extent of knowledge on incivility seen whether on their part or on part of the students.
